# Study on Modification of Poplar Wood via Composite Impregnation with Silica Sol/Melamine–Glyoxal Resin

**DOI:** 10.3390/polym15214247

**Published:** 2023-10-28

**Authors:** Mingli Liu, Xiangrui Li, Zexiu Qin, Wenbo Liu, Chunfeng Li, Lei Le

**Affiliations:** School of Materials Science and Engineering, Beihua University, Jilin 132013, China; liumingli17@163.com (M.L.); lixiangrui1209@163.com (X.L.); q15615125319@163.com (Z.Q.); liuwenbo6558@163.com (W.L.)

**Keywords:** melamine–glyoxal resin, poplar, modification, physical and mechanical properties

## Abstract

In order to overcome the defects of fast-growing poplar wood, such as low strength and poor toughness, this paper introduces a method of modifying poplar wood via impregnation with silica sol/melamine–glyoxal (silica sol/MG) resin and explores its effects on the physical, mechanical, and thermal properties of poplar wood. It was found via scanning electron microscopy that the composite modifier covered and filled the cell lumen, cell interstitial space, and cell wall pores of poplar wood. Further, infrared spectroscopy and X-ray photoelectron spectroscopy analyses confirmed that chemical cross-linking occurred between the silica sol/MG resin composite modifier and the internal groups of poplar wood and that the Si-O-Si flexible long chains introduced in the composite modifier formed a cross-linking network with poplar wood such as Si-O-Si and Si-O-C, which led to the improvement of the physical and mechanical properties and the enhancement of the thermal stability of poplar wood. The method provides a theoretical basis for the high-value utilization of fast-growing poplar wood.

## 1. Introduction

Wood is a natural renewable resource widely used in industry, agriculture, construction, and other fields [1] and is closely related to daily life. However, the global decline in forest resources has increased tension between wood supply and demand. Due to their short growth cycle and high yield, plantation species have, to a certain extent, alleviated the problem of contradictory supply and demand for domestic timber resources [2].

Fast-growing poplar wood presents challenges such as low density, low strength, easy deformation and cracking, flammability, and perishability, which limit its potential applications and utilization. In order to improve the performance of fast-growing poplar wood and extend its service life, modified treatment is needed [3].

Wood modification means treating wood using physical and chemical methods while preserving its inherent properties to enhance its resistance to dry shrinkage, wet swelling, flammability, decay, and suboptimal mechanical characteristics. This process substantially enhances wood performance, giving it novel functions and augmenting its utilization value [4]. Generally, wood is treated using physical, chemical, and combined physical–chemical methods, and common modification methods include compression densification, high-temperature heat treatment, acetylation modification, furfurylation modification, and resin impregnation modification [5].

Among them, resin impregnation modification refers to using low molecular weight, water-soluble resin impregnation treatment of wood under atmospheric pressure or vacuum pressurization. After drying and curing, water-insoluble polymers are generated inside the wood. The filling of these polymers leads to the thickening of the cell wall and the narrowing of the intercellular gap of poplar wood, making it denser and more rigid to improve the dimensional stability, mechanical strength, and fire-retardant and other properties of the wood, a kind of wood modification technology [6,7,8].

With the continuous research on impregnation modification, the combined impregnation of organic resins and inorganic materials to modify wood has become a prominent hot spot in current research. The organic–inorganic composite can reduce the amount of organic resin and fully use its encapsulation (or bonding) to immobilize the inorganic modifier to reduce the loss [9,10]. Therefore, with the synergistic effect of organic-inorganic materials, the performance of modified wood can be significantly improved, and the practical value and application areas can be further expanded.

Melamine formaldehyde resins are widely used because of their strong chemical activity, giving them a high reaction rate, thermal stability, hardness, wear resistance, and chemical resistance [11,12,13]. However, due to the release of free formaldehyde during synthesis or use, resin poses environmental pollution and potential risks to human health, thereby limiting its applications. Therefore, research scholars tried to replace formaldehyde with glyoxal to react with melamine to prepare environmentally friendly melamine–glyoxal (MG) resin for impregnating modified wood, which improves the physical and mechanical properties of wood. In addition, MG resin can fill the wood cell cavity and cell wall, and cross-linking with the wood improves the mechanical properties of the wood. These effects can improve the performance of impregnated, modified materials [14].

As an environmentally friendly inorganic material with good thermal stability and biocompatibility, silica sol can effectively penetrate wood and form a stable space structure in the pore structure of wood due to its low viscosity and small particle size [15]. Combining silica sol with MG resin impregnation to modify poplar wood, enhancing its toughness and overall performance, holds significant value in raising its added value and expanding its range of applications [16].

In our previous study, KH-560 was used to modify the surface of silica sol. The silica sol/MG resin was prepared via in situ polymerization under weakly acidic conditions using melamine, glyoxal, and modified silica sol as raw materials and NaOH as catalyst [15]. In this study, we will further use the impregnation process to prepare poplar wood-modified material and explore the effect of the composite modifier on the physical and mechanical properties of the modified material. Scanning electron microscopy (SEM), thermogravimetric analyzer (TG), infrared spectrometer, X-ray photoelectron spectroscopy (XPS), and other means of characterization were used to analyze the microstructure of poplar before and after the modification, as well as the changes in the distribution of the modifier inside the wood, thermal stability, functional groups, and other changes, to reveal the silica sol/MG resin impregnation enhancement of toughened poplar wood modification mechanism.

## 2. Materials and Methods

### 2.1. Sample Preparation

#### 2.1.1. Materials

Poplar from Jiaohe Forestry, Jilin Province, was used in this study with a density of about 0.42 g/cm^3^. The utilized samples were defect-free, devoid of cracks or knots. They were cut according to the specified testing dimensions: 300 mm × 20 mm × 20 mm (L × R × T) for bending strength, modulus of elasticity, and impact toughness tests; 20 mm × 20 mm × 20 mm (L × R × T) for wood weight gain, water absorption, and dimensional stability assessment; and 70 mm × 50 mm × 50 mm (L × R × T) for wood hardness evaluation. The poplar specimens in these three sizes were dried to a moisture content of 7–12%. For each test, 12 spare specimens were selected. The other materials required are shown in Table 1.

#### 2.1.2. Preparation of Modified Silica Sols

A total of 200 g of silica sol was added to the mixed solution of ethanol and water (volume ratio of 1:1), which was ultrasonically shaken for 30 min and poured into a three-necked flask with a water bath set to a constant temperature of 60 °C. The reaction was terminated by adding measured KH-560 dropwise during 5 h of stirring. The light blue modified silica sol solution was obtained and left to stand.

#### 2.1.3. Preparation of Silica Sol/MG Resin Composite Modifier

According to the molar ratio of M/G 1:7, a certain amount of melamine and glyoxal was added to the beaker with different mass fractions of modified silica sol (calculated as the mass percentage of MG resin). The mixture of melamine, glyoxal, and modified silica sol was ultrasonically shaken for 15–30 min to make the modified silica sol evenly dispersed in the solution. The reaction mixture was poured into a three-neck flask and adjusted to weak acidity with a mass fraction of 30% NaOH. The reaction temperature was set at 70 °C and kept for 120 min, and the silica sol/MG resin composite modifier was obtained after cooling to room temperature.

#### 2.1.4. Preparation of Silica Sol/MG Resin-Modified Poplar

The silica sol/MG resin compound modifier was dissolved in deionized water and mixed with a glass rod to form a 45% mass fraction of the impregnating solution for use. The poplar wood was impregnated using the impregnation modification process (impregnation pressure: 1.2 MPa and impregnation time: 3 h) to produce silica sol/MG resin-modified poplar wood. All specimens were sequentially dried at 60 °C, 80 °C, and 103 °C for 24 h each, following the impregnation process.

### 2.2. Testing Instruments

The instrumentation required for the test is shown in Table 2.

### 2.3. Performance Testing and Characterization

#### 2.3.1. Weight Percent Gain

The weight gain rate of the wood is calculated according to Equation (1).

(1) $$WPG=\frac{M_{1}-M_{0}}{M_{0}}\times 100\%.$$

*WPG* is weight percent gain dry weight gain rate, given in percentage (%). *M*_0_ is the absolute dry mass given in grams (g) before impregnation. Similarly, *M*_1_ is the absolute dry mass after impregnation expressed in grams (g).

#### 2.3.2. Density, Water Absorption (WA), and Dimensional Stability Tests

Density, water absorption, and dimensional stability were tested in compliance with the standards GB/T 1927.5-2021 “Test methods for physical and mechanical properties of small clear woodspecimens—Part 5: Determination of density”, GB/T 1927.7-2021 “Test methods for physical and mechanical properties of small clear woodspecimens—Part 7: Determination of water absorption”, and GB/T 1927.8-2021 “Test methods for physical and mechanical properties of small clear woodspecimens—Part 8: Determination of swelling” [17,18,19]. Dimensional stability is characterized by anti-swelling efficiency (ASE), which is calculated according to Equation (2).

(2) $$ASE=\frac{\left(L_{rs}\times L_{ts}\times L_{hs})-(L_{r0}\times L_{t0}\times L_{h0}\right)}{\left(L_{r0}\times L_{t0}\times L_{h0}\right)}\times 100\%$$

Here, *ASE* is the rate of expansion and contraction resistance expressed in percentages, %. Moreover, *L_rs_*, *L_ts_*, and *L_hs_* are the radial, chordal, and grain-wise dimensions of the specimen at a moisture content above the saturation point of the fibers expressed in millimeters (mm). Similarly, *L_rs_*, *L_ts_*, and *L_hs_* are the radial, chordal, and grain direction dimensions of the specimen when fully dry, expressed in millimeters (mm).

#### 2.3.3. Mechanical Performance Testing

Modulus of rupture (MOR), modulus of elasticity (MOE), impact toughness, and hardness were tested according to the standards GB/T 1927.9-2021 “Test methods for physical and mechanical properties of small clear woodspecimens—Part 9. Determination of bending strength”, GB/T 1927.10-2021 “Test methods for physical and mechanical properties of small clear woodspecimens—Part 10: Determination of modulus of elasticity in bending”, GB/T 1927.17-2021 “Test methods for physical and mechanical properties of small clear woodspecimens—Part 17: Determination of impact bending strength”, and GB/T 1927.19-2021 “Test methods for physical and mechanical properties of small clear woodspecimens—Part 19: Determination of static hardness” [20,21,22,23].

#### 2.3.4. SEM Inspection

Wood pieces of specific dimensions were obtained via cutting from poplar and modified poplar specimens with dimensions of (20 ± 1) mm × (20 ± 1) mm × (20 ± 1) mm. The cross-section, diameter section, and chord section of the specimens were appropriately marked, dried until a constant weight was achieved, and then set aside. The specimens were scanned via electron microscopy with gold spray treatment, and the morphology of the specimens was evaluated and analyzed.

#### 2.3.5. TG-DTG Testing

The samples were assessed for thermal weight loss at 25–800 °C, with a temperature increase rate of 10 °C/min and an N_2_ atmosphere. The injection volume was about 5 mg.

#### 2.3.6. FT-IR Detection

The specimens were crushed using a crusher and passed through a 200-mesh sieve to study the structural changes of poplar wood before and after modification. The samples were prepared using the KBr compression method. Subsequently, infrared spectral maps of both poplar and modified poplar were obtained within the wave number range of 4000–400 cm^−1^.

#### 2.3.7. XPS Detection

The specimens were crushed and passed through a 200-mesh sieve for X-ray photoelectron spectroscopy analysis. The Al-Kα target was used, the X-ray beam energy was 100 W, the grating diameter was 200 mm, the sensitivity was 350 kcps, and the vacuum of the analysis chamber was 10–8 bar.

## 3. Results and Discussion

### 3.1. Comparative Analysis of Physical and Mechanical Properties of Poplar and Modified Poplar

**Figure 1:**
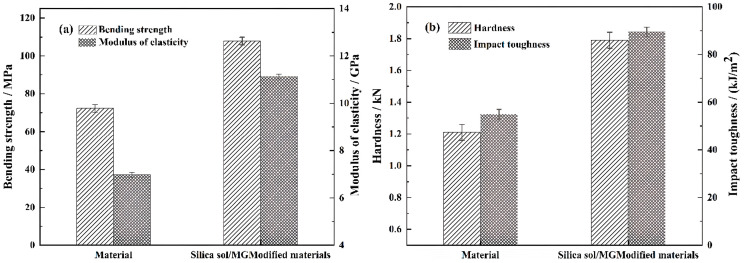
Mechanical properties of poplar and MG modified poplar: (**a**) flexural strength and modulus of elasticity as well as (**b**) hardness and impact strength.

Table 3 and Figure 1 show the test results for the physical and mechanical properties of both poplar and silica sol/MG resin-modified poplar. As seen in Table 1, after vacuum pressure impregnation treatment, the density of the modified wood is 0.53 g/cm^3^, which is 26.19% higher than that of the material. Moreover, the weight gain rate of the modified wood reaches 50%. The weight gain rate of the wood is an intuitive reflection of the amount of impregnation of the modified wood, which indicates that the wood is better filled into the wood. After the impregnation treatment with the silica sol/MG resin composite modifier, the water absorption rate exhibited a significant reduction trend. Specifically, the water absorption rate of the modified poplar was 60.56% lower than that of the poplar. This observation indicates effective diffusion and penetration of the composite modifier into the cell interstices, cell walls, and even cell cavities of the wood under pressure. As silica sol/MG resin played a good filling role, it was better covered in the cell conduit. The cell walls and cell cavities make it difficult for water to come into contact with the cellular components, thus reducing the water absorption rate at the physical level. In addition, the curing and deposition of the modifier on the inside of the wood make the pores of the wood filled, the water channels of the wood are reduced, and the moisture and water absorption rate of the modified wood are reduced. On the other hand, due to the reaction between silica sol/MG resin and hydroxyl groups in the internal chemical components of poplar wood, the number of hydroxyl groups is reduced, and the reduction in hydroxyl groups also reduces the water absorption rate of wood to a certain extent [24].

As shown in Figure 1, the physical and mechanical properties of the silica sol/MG resin-modified poplar were improved compared with the poplar, and the overall mechanical properties showed a linear growth trend. The hardness of the modified poplar was 1.79 kN, which was 47.93% higher than that of the poplar. The impact strength of the modified poplar was 89.50 kJ/m^2^, which was 62.73% higher than that of the poplar. It is because the silica sol/MG resin composite modifier is well filled in poplar wood after impregnation treatment, curing into a stable and robust cross-linking network that can share the load for the poplar wood matrix and inhibit cracking during load bearing, thus improving the mechanical properties of the wood [25]. 

**Figure 2:**
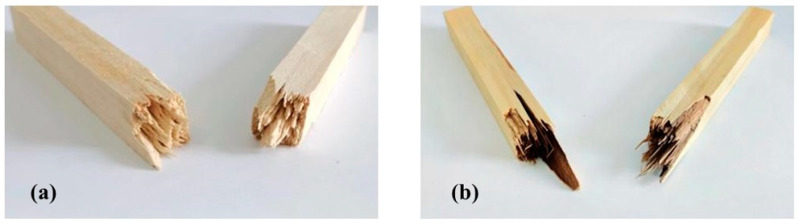
Impact failure sections of poplar and silica sol/MG resin-modified poplar: (**a**) poplar (**b**) silica sol/MG resin-modified poplar.

The pictures of poplar and modified poplar after fracture with the impact test are shown in Figure 2. From the figure, it can be seen that the impact section of the modified poplar has noticeable wood fiber pulling and pulling out compared with the poplar, which indicates that the toughness of the wood becomes better after the impregnation modification, and the toughness of the silica sol/MG resin-modified poplar is much higher than that of the poplar. In summary, it can be seen that the impact toughness of the composite-impregnated modified poplar has the most remarkable improvement.

### 3.2. Effect of Silica Sol/MG Resin Modification on the Micromorphology of Poplar Wood

**Figure 3:**
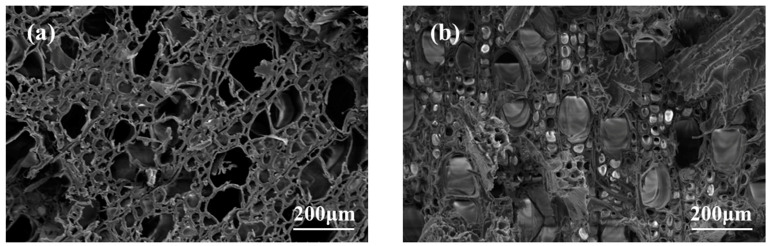
Scanning electron micrographs of the cross-section of poplar and silica sol/MG resin-modified poplar: (**a**) poplar and (**b**) modified poplar.

**Figure 4:**
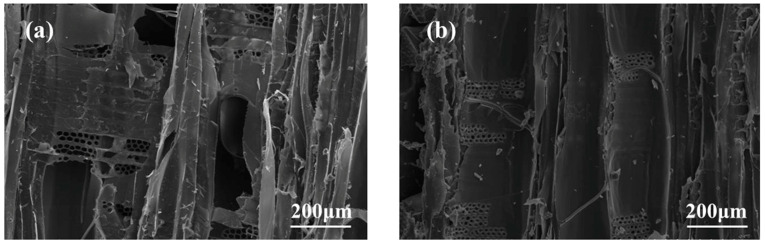
Scanning electron micrographs of the radial section of poplar and silica sol/MG resin-modified poplar: (**a**) poplar and (**b**) modified poplar.

**Figure 5:**
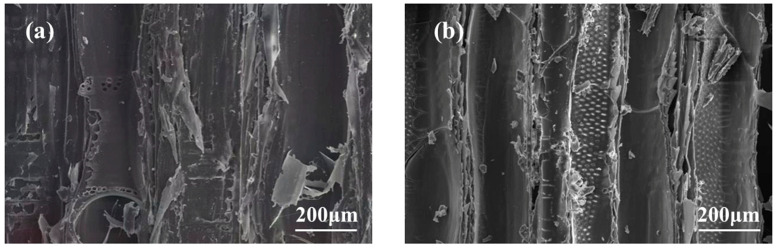
Scanning electron micrographs of tangential sections of poplar and silica sol/MG resin-modified poplar: (**a**) poplar and (**b**) modified poplar.

To study the distribution of the composite modifier within the wood cells, scanning electron microscopy was used to observe the microscopic changes in the cross-section (Figure 3), radial section (Figure 4), and tangential section (Figure 5) of both poplar and silica sol/MG resin-modified poplar. The porous structure and sparse cellular corners of the poplar are easily observed in Figure 3a, with well-defined pores and no fillers. After treatment with silica sol/MG resin composite modifier impregnation (Figure 3b), a large amount of modifier filling is evident in the cell cavities and cell interstices of the wood, which penetrates the wood and produces cross-linking and curing, making the cell structures denser to each other and thus increasing the resistance of the wood structure to external forces [26].

Figure 4a and Figure 5a show that the inner walls of the ducts and wood fiber cells in the radial and tangential sections of the poplar are smooth and flat, and the cell cavities and cell wall pores are hollow. In contrast, these channels of the modified poplar are covered and filled with modifiers, as seen in Figure 4b and Figure 5b. The tangential section of the modified poplar is mostly cured and deposited composite modifier, indicating that the composite modifier can effectively enter the poplar cells and the composite modifier can effectively enter the poplar wood cells and present a good dispersion in the wood substrate. It is stabilized inside the wood via chemical cross-linking, which leads to the overall improvement of the physical and mechanical properties of the wood [27].

### 3.3. Effect of Silica Sol/MG Resin Modification on the Thermal Stability of Poplar

**Figure 6:**
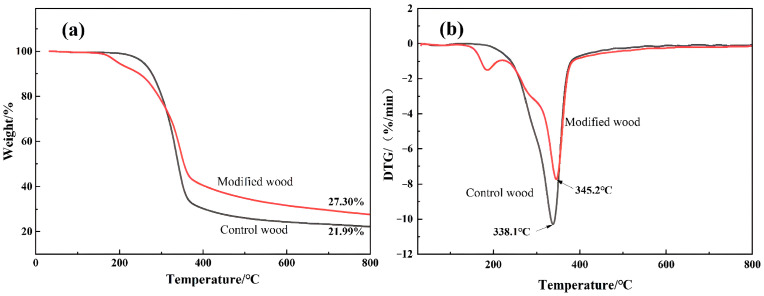
TG (**a**) and DTG (**b**) curves of poplar and silica sol/MG resin-modified poplar.

The TG-DTG curves of poplar silica sol/MG resin-modified poplar are shown in Figure 6. The TG curves show that the thermal degradation process of the poplar and modified poplar can be roughly divided into the following three stages: The first stage is the drying stage, where the temperature between 25 and 120 °C is mainly the result of mass loss caused by the evaporation of water. The second stage is the charring stage, where the temperature between 120 and 380 °C mainly corresponds to the pyrolysis of cellulose and hemicellulose in the wood and the gradual formation of char in the C-C bond in the lignin unit benzene–propane graphite structure [28,29,30]. However, during this stage, a new pyrolysis phase occurs in the modified poplar at 140–220 °C, which may be generated with glyoxal hydrate or oligomer degradation in the composite modifier. The mass loss of the poplar at this stage was about 67.65%. In comparison, the maximum mass loss of the modified poplar was 15.57% lower than that of the poplar, indicating that the addition of the modifier increased the thermal stability of the wood. The third stage, known as the calcination stage, commenced at 380 °C and extended until the end of the reaction. During this stage, both poplar and modified poplar experienced minimal mass loss, primarily attributable to the gradual degradation of the remaining components within the wood. At 800 °C, the residual carbon rate of the poplar and modified poplar was 21.99% and 27.30%, respectively, indicating that the impregnation of silica sol/MG resin composite modifier into the wood played a role in stabilizing the wood residues.

From the DTG curves, the temperatures corresponding to the maximum pyrolysis rates of the material and the modified material were 338.1 °C and 345.2 °C, respectively, and the maximum pyrolysis temperature of the modified material was 7.1 °C higher than that of the material. It is due to the chemical cross-linking reaction between the composite modifier and the cellulose and other components in the wood, which reduces the content of volatile molecules in poplar wood and thus improves the thermal stability of poplar wood-modified material. On the other hand, due to the introduction of silica sol in silica sol/MG resin, the sol has strong thermal stability, thus improving the thermal stability of silica sol/MG resin-modified material to a certain extent [31].

### 3.4. Effect of Silica Sol/MG Resin Modification on the Chemical Structure of Poplar

#### 3.4.1. FT-IR Analysis

**Figure 7:**
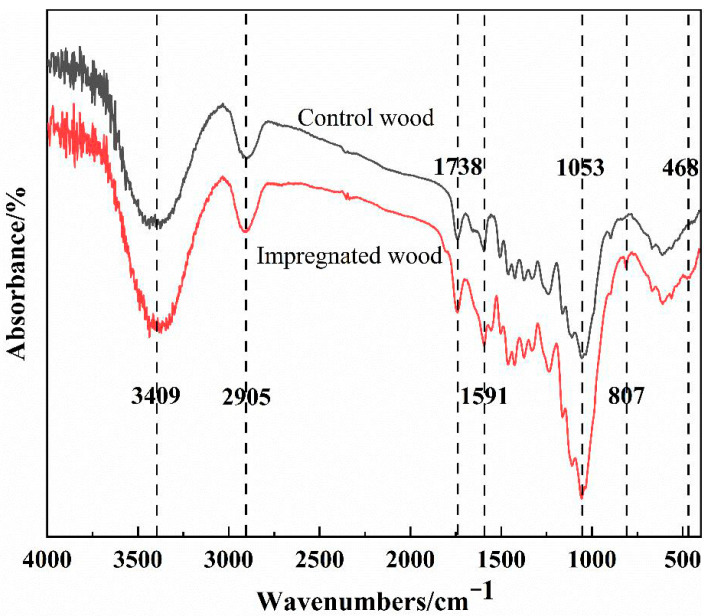
Infrared spectra of poplar and silica sol/MG resin-modified poplar.

The IR spectra of poplar and silica sol/MG resin-modified poplar are shown in Figure 7. Compared with the poplar, the expansion and vibrational absorption peaks of the hydroxyl group (-OH) and amino group (-NH_2_) in the modified wood at 3415 cm^−1^ are shifted in the direction of the low wavelengths. The intensity is weakened, and the asymmetric expansion and vibrational peaks of C-H in the methylidene-CH_2_ at 2905 cm^−1^ are also weakened [32]. On the one hand, this may be due to the decomposition of some hemicelluloses containing -CH_2_ and -OH during drying at elevated temperatures. On the other hand, the reactive groups in the modifier interacted with the hydroxyl groups of the wood, resulting in a reduction in the quantity of hydroxyl groups [33].

The most significant differences in the IR absorption profiles of poplar before and after modification were found in the range of 1800–400 cm^−1^. The absorption peak at 1738 cm^−1^ in the poplar was attributed to the stretching vibration of C=O on the acetyl and carboxyl groups of hemicellulose, while the intensity of the peak here increased in the modified poplar, indicating that more C=O bonds were formed via cross-linking between the composite modifier and the wood polymer. The decrease in wave intensity of the modified wood at 1653 cm^−1^ and 1507 cm^−1^ may be due to the hydrolysis of acetyl groups of poplar hemicellulose to acetic acid in the alkaline modifier or some degradation of lignin and carbohydrates in the modified wood [28]. Compared with the poplar specimen, the absorption peaks of the modified poplar are significantly enhanced at 1591 cm^−1^ and 1053 cm^−1^. Additionally, new absorption peaks appear at 807 cm^−1^ and 468 cm^−1^. Among them, the N-C=N bending vibration absorption peak in the melamine triazine ring structure with the deformation vibration absorption peak of the ring at 1591 cm^−1^, the antisymmetric stretching vibration peak of the Si-O-Si bond at 1053 cm^−1^, which may also be the stretching vibration peak of the new Si-O-C bond generated by the modifier and wood components, and the characteristic absorption peak of the melamine out-of-plane ring vibration with the Si-O-Si bond at 807 cm^−1^. The Si-O-Si bond symmetric stretching vibration peak at 807 cm^−1^ and 468 cm^−1^ is attributed to the bending vibration of the Si-O-Si bond. In summary, it can be seen that the silica sol/MG resin composite modifier immersed inside the wood not only acts as a physical filler but also chemically bonds with the main components of the wood cell wall.

#### 3.4.2. XPS Analysis

**Figure 8:**
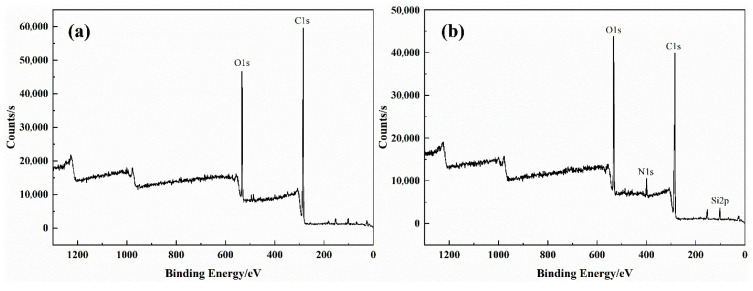
XPS spectra of poplar and silica sol/MG resin-modified poplar surface: (**a**) poplar and (**b**) modified poplar.

The full spectrum of poplar and silica sol/MG resin-modified poplar was scanned via X-ray photoelectron spectroscopy, and the results are shown in Figure 8. It can be seen from the figure that the poplar and modified poplar have strong peaks near 285 eV and 532 eV, which are the absorption peaks of C and O atoms, respectively. The chemical composition of the wood surface changed after the impregnation treatment. Moreover, the absorption peaks of Si and N atoms near 101 eV and 399 eV were significantly enhanced, indicating an increase in Si and N elements in the modified poplar, which further indicates that the silica sol/MG resin composite modifier was effectively impregnated into the wood.

**Figure 9:**
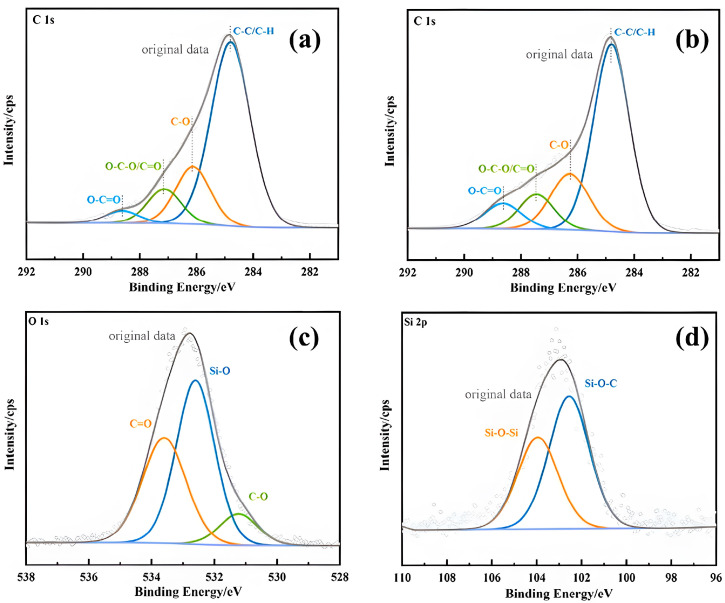
C 1s, O 1s, and Si 2p XPS spectra after peak separation of poplar and silica sol/MG resin-modified poplar: (**a**) poplar and (**b**–**d**) modified poplar.

Figure 9a,b show the XPS high-resolution spectra of the poplar and the modified poplar surface after C 1s splitting, fitted with four peaks, and the fitted data are shown in Table 4. The contents of C1, C2, C3, and C4 of the poplar were 67.06%, 18.47%, 10.69%, and 3.78%, respectively, which showed that C1 and C2 were the main binding modes of C atoms. After impregnation treatment, the chemical state of C atoms on the wood surface changed significantly. The C1 content in the modified wood decreased to 60.83%. In contrast, the C2, C3, and C4 contents increased to 19.59%, 11.00%, and 8.58%, respectively, mainly reflecting the elevated oxidation state of C, indicating that the oxygen-containing functional groups in the modified wood increased. Figure 9c,d show the XPS spectra of the modified wood surface after O 1s and Si 2p splitting. The peaks for C=O, Si-O, and C-O bonds in the O 1s spectrum of Figure 9c are at 533.6 eV, 532.6 eV, and 531.23 eV, respectively. Moreover, the peaks at 103.95 eV and 102.55 eV in the Si 2p spectrum in Figure 9d correspond to the Si-O-Si and Si-O-C bonds, respectively. The results show that the increased oxygen-containing functional groups in the modified wood and the emergence of Si-O-C bond peaks affirm the chemical cross-linking of the silica sol/MG resin composite modifier with the internal wood groups [33,34,35]. This finding aligns with the FT-IR analysis results of both the poplar and modified poplar discussed earlier.

The FT-IR and XPS analyses indicate chemical bonding between the silica sol/MG resin composite modifier and wood. Wood is mainly composed of cellulose, hemicellulose, and lignin. A large number of hydroxyl groups in the structure provide active sites for cross-linking between the composite modifier and the wood cell wall. On the one hand, the aldehyde group in the silica sol/MG resin composite modifier is nucleophilic to the hydroxyl groups on the cellulose and other components. The aldehyde group of one molecule in the modifier will react with the hydroxyl groups of two molecules on the cellulose and other components to form a stable ring structure, reflected in the improvement of the mechanical properties of the modified poplar. The flexural strength, elastic modulus, and hardness of the modified poplar were increased by 49.25%, 59.46%, and 47.93%, respectively, compared with the poplar. On the other hand, the introduction of Si-O-Si flexible long chains in the composite modifier formed cross-linked networks such as Si-O-Si and Si-O-C with wood, which improved the impact toughness of the modified poplar by 62.73% compared with the poplar.

## 4. Conclusions

In this study, building upon previous research on silica sol/MG resin, the effect of the silica sol/MG resin composite modifier on various properties of poplar wood was investigated. These properties included weight gain, water absorption, dimensional stability, flexural strength, flexural modulus of elasticity, and impact toughness. The investigations were conducted using modified poplar wood prepared through a vacuum-pressurized impregnation process. On this basis, the microstructure, thermal stability, and chemical structure of poplar wood were characterized and analyzed via SEM, TG-DTG, FT-IR, XPS, and other testing methods, and the study results are as follows:

(1) Comparative analysis of the properties between poplar and modified poplar revealed improvements in the physical and mechanical properties of silica sol/MG resin-modified poplar compared to the unmodified poplar. Moreover, the mechanical properties displayed a consistent linear growth trend. Compared with the poplar, the density of silica sol/MG resin-modified poplar increased, and the water absorption rate decreased. In addition, the MOR, MOE, hardness, and impact toughness increased by 49.25%, 59.46%, 47.93%, and 62.73%, respectively. Combining the impact damage cross-sectional profiles of the poplar and the silica sol/MG resin-modified poplar, it can be concluded that the impact toughness of the wood improved the most after the composite impregnation modification;
(2) SEM observation showed that the cell cavities, cell interstices, and cell wall pores of the modified poplar were covered and filled by the compound modifier, and the filling effect was good;
(3) The results of TG-DTG analysis showed that the maximum pyrolysis temperature of the modified poplar was 345.2 °C, which increased by 7.1 °C compared with the poplar, and the residual char rate of poplar wood increased from 21.99% to 27.30%, indicating that the impregnation treatment improved the thermal stability of the wood;
(4) FT-IR and XPS results showed that the silica sol/MG resin composite modifier was physically filled into the wood and chemically bonded with the main components of the wood cell wall. The increased oxygen-containing functional groups in the modified wood and the appearance of Si-O-C bond peaks confirmed that the silica sol/MG resin composite modifier was chemically cross-linked with the internal wood components. It was further validated by the increase in oxygen-containing functional groups and the appearance of Si-O-C bond peaks in the modified poplar, confirming the chemical cross-linking with the internal wood groups. The penetration of the silica sol/MG resin modifier into the wood and the cross-linking and curing increase the structural resistance of the wood to external forces, thus improving the dimensional stability and mechanical properties of poplar wood.

## Figures and Tables

**Table 1:** Experimental materials.

Reagent Name	Specification	Manufacturers
Melamine	Analysis of pure	Tianjin Guangfu Fine Chemical Research Institute, Tianjin, China
Glyoxal	40 wt%, Industrial grade	Tianjin Comio Chemical Reagent Co., Tianjin, China
Sodium hydroxide	Analysis of pure	Tianjin Damao Chemical Reagent Factory, Tianjin, China
Anhydrous ethanol	Industrial grade	Tianjin Damao Chemical Reagent Factory, Tianjin, China
Distilled water	-	Laboratory Homemade, Jilin, China
Silicone sol	Solid content 30%, average particle size 8–15 nm	Jinan Yinfeng Silicon Products Co., Jinan, China
Silane coupling agent KH-560	97%	Shandong Yousuo Chemical Technology Co., Linyi, China

**Table 2:** Experimental equipment.

Instrument Name	Model	Manufacturers
Push table saw	MJ6132B	Foshan Shunde District Xinma Woodworking Machinery Equipment Co., Foshan, China
Electric heating blast drying oven	DHG-9075-III	Shanghai Yiheng Technology Co., Shanghai, China
Universal mechanical testing machine	DWD-100E	Jinan experimental group limited times group, Jinan, China
Ultrasonic cleaner	DSA50-JY2	Hangzhou Desen Ultrasonic Equipment Co., Hangzhou, China
Impact testing machine	JB-300B	Jinan pilot gold group Co., Jinan, China
Vacuum pressurized impregnation tanks	VPI250	Shenyang Weike Vacuum Technology Co., Shenyang, China
Electronic analytical balance	FA2004	Tianjin Tianma Hengji instrument Co., Tianjin, China
Vernier calipers	01120028	Shanghai Taikai Gauges Co., Shanghai, China
Fourier Transform Infrared spectrometer	WQF-510A	Beijing Ruili Analytical Instrument Co., Beijing, China
Thermogravimetric analyzer	TG 209 F3	Germany Netzsch Instrument Manufacturing Co., Selb, Germany
X-ray photoelectron spectrum analyzer	ESCALAB250Xi	Thermo USA Ltd., Waltham, MA, USA
Environmental scanning electron microscope	Quanta200	Phillip (PEI), Amsterdam, The Netherlands

**Table 3:** Physical properties of poplar and modified poplar.

Specimen	Density/g cm^−3^	WPG/%	WA/%	ASE/%
Poplar	0.42	-	130.28	-
Modified poplar	0.53	50.00	51.38	42.03

**Table 4:** Fitting data of C 1s splitting on the surface of poplar and silica sol/MG resin-modified poplar.

Sample	C1/% C-C/C-H	C2/% C-O	C3/% O-C-O/C=O	C4/% O-C=O
Poplar	67.06	18.47	10.69	3.78
Modified poplar	60.83	19.59	11.00	8.58

## Data Availability

The data presented in this study are available on request from the corresponding author.
